# Accounting for variations in ART program sustainability outcomes in health facilities in Uganda: a comparative case study analysis

**DOI:** 10.1186/s12913-016-1833-4

**Published:** 2016-10-18

**Authors:** Henry Zakumumpa, Sara Bennett, Freddie Ssengooba

**Affiliations:** 1 Makerere University, School of Public Health, Kampala, Uganda; 2 Johns Hopkins University, Bloomberg School of Public Health, Baltimore, MD USA

**Keywords:** Sustainability, HIV treatment, Health systems, ART scale-up, Implementation, Case-study

## Abstract

**Background:**

Uganda implemented a national ART scale-up program at public and private health facilities between 2004 and 2009. Little is known about *how* and *why* some health facilities have sustained ART programs and why others have not sustained these interventions. The objective of the study was to identify facilitators and barriers to the long-term sustainability of ART programs at six health facilities in Uganda which received donor support to commence ART between 2004 and 2009.

**Methods:**

A case-study approach was adopted. Six health facilities were purposively selected for in-depth study from a national sample of 195 health facilities across Uganda which participated in an earlier study phase. The six health facilities were placed in three categories of sustainability; High Sustainers (2), Low Sustainers (2) and Non- Sustainers (2). Semi-structured interviews with ART Clinic managers (*N* = 18) were conducted. Questionnaire data were analyzed (*N* = 12). Document review augmented respondent data. Based on the data generated, across-case comparative analyses were performed. Data were collected between February and June 2015.

**Results:**

Several distinguishing features were found between High Sustainers, and Low and Non-Sustainers’ ART program characteristics. High Sustainers had larger ART programs with higher staffing and patient volumes, a broader ‘menu’ of ART services and more stable program leadership compared to the other cases.

High Sustainers associated sustained ART programs with multiple funding streams, robust ART program evaluation systems and having internal and external program champions.

Low and Non Sustainers reported similar barriers of shortage and attrition of ART-proficient staff, low capacity for ART program reporting, irregular and insufficient supply of ARV drugs and a lack of alignment between ART scale-up and their for-profit orientation in three of the cases.

**Conclusions:**

We found that ART program sustainability was embedded in a complex system involving dynamic interactions between internal (program champion, staffing strength, M &E systems, goal clarity) and external drivers (donors, ARVs supply chain, patient demand). ART program sustainability contexts were distinguished by the size of health facility and ownership-type. The study’s implications for health systems strengthening in resource-limited countries are discussed.

## Background

Globally, there is growing critical interest attached to the goal of universal access to HIV treatment. In September 2014, UNAIDS unveiled the 90-90-90 global targets, part of which aim at enrolling 90 % of those with HIV infection on sustained antiretroviral therapy (ART) by 2020 [1]. The Sustainable Development Goals (SDGs) announced in September 2015 re-affirmed universal access to HIV treatment in the new international development agenda [2]. In November 2015, WHO released treatment guidelines requiring that all diagnosed as HIV positive be initiated on ART regardless of disease stage [3].

Attaining these global targets and treatment guidelines in the developing world, particularly in Sub Saharan Africa, will depend substantially on the capacity of health systems to sustain and expand ART scale-up [4, 5]. Global Health Initiatives such as PEPFAR and The Global Fund, which supported the rapid expansion in ART coverage in Sub Saharan Africa, have increasingly recognized the importance of health systems strengthening in the attainment of ART scale-up goals [6, 7].

Uganda started implementing a national ART scale-up program in June 2004 with reliance on external donor support. HIV service delivery was scaled-up from tertiary to primary care facilities and from public to non-public facilities [8, 9]. The national ART scale-up program was also implemented in private-for-profit health facilities [40].

The nature of donor support to enable health facilities to commence ART delivery involved supply of free antiretroviral (ARV) drugs, provision of diagnostic equipment such as CD4 machines, laboratory capacity support and ART standard-of-care training. In Uganda, donors finance over 80 percent of ART program costs [10]. This support was provided to health facilities under time-limited project grants. In the case of PEPFAR, the predominant donor, support was channeled through larger intermediary organizations known as ‘implementing partners’ on 5-year grant cycles [11]. Increasingly, the primary funders of HIV/AIDS services in Uganda are seeking to ensure the efficiency, sustainability and country of ownership of ART programs [4–7]. This brings the question of the sustainability of ART in countries like Uganda to the top of the policy agenda.

There are numerous studies reporting on initial implementation of ART scale-up [12–15] and associated clinical outcomes [16–18]. However, little is known about *how* and *why* some health facilities have sustained ART and why some have not sustained these interventions over the past 12 years. What conditions, contexts or processes are conducive for the long-term sustainability of ART at the organizational level of ART providers? [19]. The objective of the study was to identify facilitators and barriers to the long-term sustainability of ART delivery at health facilities in Uganda which received initial grant funding for ART start-up between 2004 and 2009.

The term ‘sustainability’ is defined in varied ways in different content fields [21, 22, 25]. Within the literature on health program sustainability, there are two dominant strands in the way sustainability is defined. On one hand, it has been defined as program continuation of a newly introduced intervention within an organization after initial implementation efforts have ended [20–22]. On the other hand, sustainability has been defined as ‘institutionalization’ or the extent to which a new intervention is integrated in the organizational routines of the host implementing agency [22–24]. We adopted Proctor et al. [25] s’ definition which unifies these two dimensions. They define sustainability as ‘the extent to which a newly implemented treatment is *maintained* or *institutionalized* within a service setting’s ongoing, stable operations’ (emphasis ours) [25]. This study aligns with the literature suggesting that sustainability is not an either/or phenomenon but one assessed along a continuum or levels of sustainability [20, 26, 28].

There is a paucity of research analyzing the long-term sustainability of ART provision in Uganda. However, comparative case studies examining intervention sustainability outcomes other than ART have been conducted in other fields. Savaya et al. (2009) conducted a comparative case study of six projects in Israel which operated between 1980 and 2000 to assess why some projects were sustained while others were not. They found that the human factor in terms of the leadership of the host organization compared to factors such as availability of donor funding explained the difference [26]. Wright [27] investigated the reasons why four rural primary care programs in the United States survived 30 years after implementation and found that having program champions, organization flexibility and community integration were key. LaPelle et al. [28] examined 77 tobacco treatment programs in the United States after termination of funding following a state recession and found that re-defining the scope of services and adopting alternative financing strategies distinguished between sustained and non-sustained programs. Stolldorf DP (2013) conducted a comparative case study of four hospitals with the highest and lowest scores for sustaining a nursing intervention and concluded that certain contexts and processes facilitated program sustainability in hospitals in the United States [29]. Like most of the above studies, this study is situated within the framework by Shediac-Rizkallah and Bone [20] which posits that health program sustainability is potentially influenced at the (i) programmatic, (ii) organizational and (iii) broader environment levels.

The results reported here form part of a larger study investigating the sustainability of ART programs in Uganda with regard to the determinants of sustainability, institutionalization outcomes and an exploration of ART provider contexts [30, 31].

## Methods

### Research design

A case-study design was adopted. This involved both qualitative and quantitative approaches to data collection and analysis.

### Cases selection

The cases were identified through a 2-stage process. In the first phase, a sample of 195 (out of 394) health facilities accredited to deliver ART between 2004 and 2009 were enrolled into a survey that assessed ART institutionalization using Level of Institutionalization Scales (LoIn) scales (Goodman, 1993) [23]. A 45-item questionnaire measured institutionalization based on four ‘sub- systems’ theorized to make up an organization (Production, Maintenance, Supportive, Managerial) assessed against two levels of institutionalization; routines (lower) and niche saturation (higher) A summative score was determined for each of the 195 health facilities [30]. In the second phase, six health facilities were purposively selected for in-depth study. The selected health facilities were grouped into three categories; two facilities with highest scores (High Sustainers), two facilities with lowest scores (Low Sustainers) and two facilities that stopped providing ART (Non-Sustainers) [32, 33].

The outcome of the selection of the cases based on those with the highest and lowest ART institutionalization scores, allowed us to explore ART program sustainability at different levels of care of the Ugandan health system [34, 35]. The results from the first study phase showed variations in institutionalization scores by level of care. On average, hospitals had higher institutionalization scores than health centers. HS-001 and HS-002 represent hospital-level providers compared to LS-001 and LS-002 which are mid-size health centers. NS-001 and NS-002 were smaller health centers. (Table 1).

**Table 1:** Art program characteristics of selected health facilities

Case category	Cases acronym	0wnership	Level of institutionalization score (Out of 32)	Patient numbers (June 2010)	Patient numbers (June 2015)	Art program staff size	Rural/Urban setting
High sustainers	HS-001	PUBLIC	26.8	9,540	24,408	53	URBAN
	HS-002	PNFP	26	2,556	4,337	63	URBAN
Low sustainers	LS-002	PFP	3.9	84	19	1	URBAN
	LS-001	PUBLIC	2.7	146	458	2	PERI-URBAN
Non-sustainers	NS-001	PFP	ART Discontinued April 2013	11	0	ART Discontinued April 2013	RURAL
	NS-002	PFP	ART Discontinued January 2014	324	0	ART Discontinued January 2014	RURAL

*Key: PNFP* Private Not for Profit*, PFP* Private for Profit

The six cases reflect the three major health facility ownership categories in Uganda namely; Public (LS-001), Private for Profit (LS-002, NS-001 and NS-001) and Private Not for Profit (HS-002). The selected cases had an appropriate urban/rural mix. Three of the cases were based in urban towns of Uganda compared to the other three that were based in rural areas. All selected cases were accredited ART sites suggesting a minimum level of service delivery and infrastructural capacity at the time of accreditation.

### Data collection methods

Case-study designs rely on multiple sources of data to gain richness, in depth analysis and data triangulation [36, 37]. To this end, (1) Semi-structured interviews were conducted with at least three respondents per case (*N* = 18) (2) The quantitative data collection included two instruments*.* One was a survey that generated quantitative data on ART program characteristics for each of the cases (*N* = 6). The second tool was the Level of Institutionalization (LoIn) scales which measured the extent of institutionalization of ART programs (*N* = 6) [30]. (3) On-site observation notes of program characteristics and processes at each of the cases were examined (4) Documentary analyses of grey literature in respect to the cases, such as ART program evaluation reports and websites were scrutinized to augment respondent data. Data were collected between February and May 2015 by two authors and four research assistants who were experienced in data collection in ART-providing organizations.

### Interview procedure

An interview guide was constructed based on factors identified in the literature as potentially influential on health program sustainability. The selected factors were most consistent with those identified in the review article by Scheirer MA and Dearing JW [22]. The open-ended nature of the interview guide allowed us to elicit responses from interviewees regarding the facilitators and barriers to ART program sustainment at their sites from the interviewees’ perspectives. When sustainability factors or attributes contained in the interview guide were not spontaneously raised by interviewees, probing was done to cover the remaining attributes.

We sought interviewees who had the longest ART program experience at their sites, particularly those who had been in service during the pilot phase of ART delivery at their sites. As a first step, the ART Clinic heads were contacted and requested to have their health facilities voluntarily participate in the study. All six cases agreed to participate in the study.

The ART clinic heads were then asked to nominate interviewees who met the criteria. Voluntary consent to participate of the nominated interviewees was sought. They then signed a written consent form agreeing to be interviewed for the study. Interviews were typically conducted in the respondents’ offices at the respective health facilities and lasted between 30 and 45 min. The interviews were conducted in the English language, between February and June 2015. At least three interviewees, per case, were recruited (*n* = 18).

During the interview, participants were asked to describe program characteristics such as the number of patients currently enrolled on ART and the range of HIV treatment services offered. Interviewees were then asked to describe how the ART program at their site had evolved since the initial phase of implementation. We deliberately began with an open-ended dialogue to elicit responses from interviewees regarding the facilitators and barriers to ART program sustainment at their sites from the interviewees’ perspectives. When sustainability attributes contained in the interview guide were not spontaneously raised by interviewees, probing of those attributes followed.

The interviews were recorded using an electronic recorder. The audio recordings were transcribed and stored on a password-protected computer. The authors listened to the audio recordings multiple times to ensure accuracy in transcription of the interviews.

### Data analysis

#### Qualitative data

As a first step, two authors read through the interview transcripts separately to identify themes emerging from the interview responses under ART program sustainability barriers and facilitators with respect to each of the six cases and subsequently across the cases [36, 38]. The authors sought convergence in the interpretation and the assignment of codes and themes with respect to facilitators or barriers to ART program sustainability.

In the second stage, the authors compared the themes emerging from the interviews against those in the initial coding scheme. The initial coding scheme was constructed based on factors identified in the review article by Scheirer MA and Dearing JW [22]. The codes were then methodically grouped under the three over-arching themes of the study. For ease of comparison of the cases, themes emerging from each individual case and across the cases were summarized in a two-column table [28, 39]. In the third stage, codes which were not adequately captured by the initial coding scheme or those which emerged inductively were grouped into categories to enable generation of new themes which were jointly agreed upon by the authors through consensus. Interviewee data were triangulated with other information sources such as questionnaire data and document review such as donor project evaluation reports involving the cases.

#### Case-study comparative analyses

A case description was constructed for each of the cases based on questionnaire data, provider interviews and documentary evidence to gain an understanding of the operational context of each of the cases. In the first instance, with-in case analyses was completed for each of the cases to assess facilitators and barriers of ART program sustainability based on the three principal sources of data which were processed into text data to facilitate thematic analyses. Across-case analyses were conducted for each of the three case categories to assess concurrence or divergence of the emerging facilitators and barriers to ART sustainability.

#### Quantitative data

Quantitative data were extracted from a self-administered questionnaire filled with respect to the cases. We compared closed-ended responses relating to ART program characteristics to explore distinguishing features within and across the cases (Table 1). The second questionnaire sought to measure the extent of institutionalization of ART programs at each of the cases. We compared the quantitative scores assigned to the cases computed from the descriptive statistics generated from each of the cases.

#### Mixed-methods integration

The study was conducted in two consecutive phases [38]. The results of the questionnaire measuring the level of institutionalization of ART programs at 195 health facilities [30] were used to purposively select six cases for in-depth study [38]. In the results under Section A, a case description for each of the six cases was constructed based on quantitative data (questionnaire) and qualitative (interviewee) data. In the results, under Section B, the across-case comparisons of ART program characteristics are based on quantitative data in Table 1. We draw upon the qualitative data (in Section C) to explain the significance of the differences reflected in Table 1 in Section B of the results. Additionally, in our qualitative findings in Section C, we cite instances where there is triangulation of data sources with quantitative data in Section A. Full integration of qualitative and quantitative data was done in our overall interpretation of the findings in the Discussion.

## Results

The findings from this study are presented in three sections. In the section A, a summary profile of each of the six cases is presented. The comparative case study analysis findings are presented in subsequent sections representing the three groups of factors influential on program sustainability [20];

- ART Program characteristics generated from questionnaire data (Section B).
- Qualitative analysis of barriers and facilitators to sustaining ART programs at i) Organizational and ii) broader environment levels (Section C).

## Section A

### Case descriptions

HS-001 is a public hospital located in an urban town of South-western Uganda. The ART clinic is a specialized unit within a large hospital complex. In 2004, the hospital implemented an ART scale-up program with donor support. HS-001 had the highest ART institutionalization score in a national sample of 195 health facilities.

HS-002 is an old mission hospital in Uganda located in Uganda’s capital. The ART clinic is a specialized unit within a large hospital complex. The hospital implemented an ART scale-up program in 2005 with donor funding. HS-002 had the second highest ART institutionalization score.

LS-001 is a public, Health Centre IV, located in a peri-urban setting of East Central Uganda. The Health Center serves a geographical area equivalent to a county or Health Sub-district. This health facility had the lowest ART institutionalization score in a national sample of 195 health facilities.

LS-002 is a mid-size, private clinic, in an urban town in Central Uganda. In 2009, the clinic benefited from a USAID-funded project to initiate ART delivery through site accreditation support, ART workforce training, and provision of laboratory infrastructure such as a chemistry analyzer and autoclave and on-site support supervision. This clinic has the second lowest ART institutionalization score

NS-001 is a private employee clinic located in a rural setting in Hoima district in Mid-western Uganda. The Clinic is equivalent in size to a Health Centre III. It caters to 800 families on a Tea Estate. In 2009, it benefited from USAID funding to enable it commence ART services through site accreditation support, ART workforce training and on-site support supervision. The Clinic discontinued ART delivery in January 2014.

NS-002 is a private clinic located in a remote part of Kyenjonjo district in western Uganda. In 2009, it benefited from USAID funding to initiate ART provision through site accreditation support, ART workforce training and on-site support supervision. The clinic discontinued ART delivery in April 2013.

## Section B

### ART program characteristics

Closed-ended responses to questions inquiring into the ART program characteristics of the six cases are presented and then compared across the cases.

#### Range of HIV services offered

Questionnaire data revealed that HS-001 and HS-002 offered four ART intervention components namely (i) Adult ART (ii) Pediatric ART (iii) Prevention of mother to Child Transmission (PMTC) (iv) Tuberculosis co-infection management as at June 2015.

LS-001 and LS-002 indicated that they offered one type of HIV service,that of Adult ART, whereas both NS-001 and NS-002 indicated they no longer offered ART services.

High Sustainers reported offering a wider range of HIV services compared to Low sustainers with the subsequent qualitative analysis suggesting that patients preferred ART clinics with a broader ‘menu’ of services which in turn influenced patient retention rates by providers.

#### Patient volumes

Table 1 shows that between March 2010 and April 2014, the cumulative number of patients increased in all cases, except for LS-002 where the patient numbers decreased from 84 to 19. High Sustainers had significantly higher patient loads compared to Low sustainers.

We found that difference in health facility size alone did not account for disparities in patient volumes across the cases. Qualitative data revealed that High Sustainers sought to retain or grow patient numbers to align with performance targets agreed with funders. Conversely, Low and Non-sustainers (three out of four cases) sought to deliberately cap patient numbers to align them with their service delivery capacity and their for-profit goals.

#### ART program staffing strength

Table 1 shows that High sustainers reported a higher number of ART program staff compared to Low Sustainers. Low Sustainers had a higher staff: patient ratio compared to High Sustainers. This may suggest that ART workforce productivity was higher in the High sustainers. Subsequent interviewee data revealed that ART program staffing strength, within the cases, was partly influenced by the human resources management strategies adopted.

#### ART program leadership

The heads of the ART programs at HS-001 and HS-002 had been in their positions for a period of at least 7 years. In contrast, the ART program heads of both ‘Low sustainers’ had been in their positions for a period ranging between 1 and 3 years. The results suggest that stability in program leadership is an attribute of interest in the analysis of facilitators and barriers to ART program sustainment.

#### ART program institutionalization scores

Level of Institutionalization scales adapted from Goodman (1993) [23], were used to compute and compare ART program institutionalization scores in the cases. HS-001 had the highest overall ART institutionalization score of 26.8 out of a maximum score of 32. HS-002 had the second highest score at 26. LS-001 had the lowest ART institutionalization score at 2.7 followed by LS-002 at 3.9. Both NS-001 and NS-002 discontinued ART in 2013 and 2014 respectively.

## Section C

### Qualitative analysis of facilitators and barriers to ART program sustainment

This section presents results of the qualitative interviews with participants with regard to facilitators and barriers of ART program sustainability.

## Organizational context factors

### Sustainability facilitators linked to the organizational climate of the ART provider

#### Diversifying funding sources

HS-001 and HS-002 reported a similar strategy of diversifying funding sources to sustain ART delivery over the past 10 years. They both reported seeking and attracting supplemental funding from multiple external donors. For instance, HS-002 mobilized resources for a new building to house the new ART clinic from external resources. Both cases revealed that they had been able to survive project grant cycles over the last 10 years which they attributed to adopting a deliberate strategy of diversifying funding streams.

*“We had different partners supporting different arms of HIV treatment to try and pull from different directions. We had one donor supporting us on staff salaries, the Ministry of Health providing us with drugs and another donor supporting us on the patient data base and an individual donor giving us multivitamins”* [Interviewee 1, HS-001].

*“When you have two taps pouring water into the same tank, when one tap loses, another will continue to bring in water”* [Interviewee 2, HS-002].

Interviews with Low and Non sustainers revealed that they had not been able to attract new grant funding for ART delivery since initial ART implementation. This finding was triangulated with questionnaire data where High sustainers reported five grants in the last 10 years compared to one grant in both cases in the Low sustainers’ category.

#### ART program monitoring and evaluation tools

High sustainers associated their ability to attract multiple grants from funders with maintaining robust monitoring and evaluation systems for their ART programs. High Sustainers indicated that they relied on time-limited grants for ART delivery ranging between 2 and 5 years. Successor grant funding was said to be dependent on attaining program outcomes such as growing patient numbers and reducing lost-to -follow-up cases. These benchmarks were assessed based on outcomes data generated from reporting tools such as patient information systems and maintaining ART patient registers.

*“We rely on our M & E electronic data-base to assess our own performance and to report to funders”* [Interviewee 3, HS-001].

Although monitoring and evaluation emerged strongly as a facilitator of ART program continuation among High Sustainers, conversely, it was reported as a barrier to program sustainment among Low and Non Sustainers. Low Sustainers reported that national ART guidelines required them to maintain reporting tools such as patient registers and clinical outcomes reports which they couldn’t keep up with due to low staffing capacity. The available ART workforce were said to be swamped with routine ART service delivery without spare time for manually filling hard-copy ART program reporting tools. Another constraint cited were the costs associated with patient follow-up.

*“And yet you had to fill these (M& E) forms because these drugs are followed up. We don’t even have adequate physical space for the increased clients and even for storing all these M & E records. One book (ART register) can take up an entire desk!”* [Interviewee 1, LS-002].

*“You find a patient has come from a district hundreds of Kilometers away. He wants to get drugs from here but following them up would be a problem. So, what we agreed with my staff is to handle clients who are within a 5 kilometer radius”* [Interviewee 3, LS-001].

Delays in ART program reporting were associated with ARV drugs stock outs. Interviewees indicated that requisition for the free ART commodities from the national medicines supplier was based on ART program data. Documentary evidence supported our findings. A donor report authored in 2014 indicates that LS-001 faced constraints in delivering on its ART program reporting mandates and benefited from an intervention to strengthen capacity for ART program reporting.

ART program reporting emerged as a key distinguishing feature between the categories of cases. High Sustainers prioritized ART program reporting by maintaining ART program reporting systems compared to Low and Non Sustainers where ART program reporting was not as highly prioritized that influenced program sustainability outcomes.

### The role of internal program champion

The majority of participants in HS-001 and HS-002 reported the presence of an internal program champion who was described as an influential figure within the implementing organization, instrumental in fostering the sustainability of the intervention within the facility. The program champion was perceived to have been instrumental in mobilizing resources to sustain the ART program from within and outside the health facility. The program champion was also described as key in providing leadership and overall direction to the rest of the clinic staff.

*“She writes grants and approaches donors. You know some donors work with individuals. She is constantly planning for the clinic. She is a good motivator too and inspires us to cope with the heavy workload”* [Interviewee 3, HS-002].

In contrast, Low and Non Sustainers did not report an internal program champion during the interviews even when they were probed on the subject.

*“We didn’t have such a person in our organization. I don’t believe that even the facility in-charge then played such a role”* [Interviewee 2, NS-001].

Interview data were triangulated with data extracted from the two self-administered questionnaires which were consistent with this finding.

### Barriers to ART program sustainment relating to Organizational context

There was a high concurrence in perceived barriers to ART program sustainability reported by participants in both Low Sustainers and Non-Sustainers categories.

#### ART scale-up and the organizational goals of ART providers

Participants from across Low and Non-Sustainers categories reported that ART program scale-up was often at odds with their organizational goals. The proprietor of LS-002 reported that their clinic targeted clients in the high-end market segment. The introduction of donor-supported ART services at the clinic was reported to have attracted a broader socio-economic class of patients which was at odds with their goal of providing an exclusive service that protected their clients’ privacy. In addition, donor-supported ART services at the private health facilities were reported to have attracted long patient queues which ‘crowded out’ other health care services.

*“People who come to my facility are of a high class. They are fearing or are uncomfortable being in a government facility. That is the class which I wanted but then as time went on other lower classes started coming in. We were handling most of the high class people because we want people who want privacy”* [Interviewee 1, LS-002].

Both of the Non-sustaining providers were private employee clinics located in rural settings. The participants at these health facilities reported that the introduction of donor-supported ART services attracted non-primary patients from the neighboring community which stretched their service delivery capacity and negatively affected the satisfaction of their primary clients. They expressed the constraint of being unable to turn away patients due to the service obligations associated with receiving public ARV drugs. The burden of these obligations and indirect costs contributed to the eventual discontinuation of ART services.

*“You see with ART we didn’t charge because we were getting public drugs and couldn’t stop others from coming but then the long queues were a problem”* [Interviewee 2, NS-002].

#### For-profit orientation and ART program sustainment

Interviews with providers with a for-profit orientation (3 out of 4) revealed that donor-supported ART scale-up programs were perceived as not ‘profitable’. This was in relation to the financial investment needed, on the part of providers, to deliver them to scale.

*“For me my aim is to make profit. Donors should support us with salaries for staff who run ART services. Otherwise, it becomes hard for the private clinics to sustain the services”* [Interviewee 1, LS-002].

*“Most private health facilities fear to offer free services because there are no other benefits”* [Interviewee 3, NS-001].

NS-001 revealed that after the initial donor supported them to secure site accreditation status, they started receiving free supplies of ARV drugs from the government medicines supplier and commenced ART delivery. However, ARV drug supplies became irregular and the company was frequently compelled to step in and buy drugs during stock-out events. When the supply chain bottlenecks became chronic, the company couldn’t sustain the financial commitment and discontinued ART altogether and referred patients to other providers in the district.

Interviewee data suggesting that for-profit providers were reluctant to support long-term delivery of ART at their clinics because of the implicit need to secure the services financially, was triangulated with document review evidence in an End-of-Term donor report:

*“Company clinics were reluctant to provide long term HIV and TB services which were expensive and yet with no direct returns”* Page 22 [40].

Our findings suggest that the decision to discontinue ART delivery in NS-001 and NS-002 was partly as a result of the reluctance of the parent companies to shoulder the burden of long-term delivery of ART at these private sector facilities in the event of donor or government failure to guarantee support or failure to secure the supply chain for ARV drugs and other commodities. Companies were more content to provide primary care services to their employees compared to committing themselves to chronic conditions such as HIV management.

#### ART workforce barriers to ART program sustainability

Human Resources for Health constraints emerged as an important barrier to the sustainability of ART programs across the Low and Non sustainer categories. Participants from these cases indicated they had staffing shortages and that the introduction of donor-supported free ART services attracted large patient volumes which outstripped their service delivery capacities.

*“And then the number of clients increased and even my staff could no longer handle the numbers. You know in a private clinic you can come in and find that there is only one clinical officer and one nurse on duty. And yet there were other services. I could no longer handle. In fact, I stopped. I had to refer patients to other providers”* [Interviewee 1, LS-002].

In addition, participants from all private clinics in the Low and Non-sustainers categories, reported that their inability to offer permanent terms of service, coupled with low salary levels, resulted in the attrition of their ART-proficient staff.

*“Staff are not permanent and we employ them on temporary basis. I always tell my staff “you are here on temporary basis. If you get a job somewhere and its very good. You want me to recommend you?, No problem.”. You lose people who you have trained and imparted all those skills upon”* [Interviewee, 1, LS-002].

The finding that for-profit health facilities faced challenges retaining their ART-proficient staff was triangulated with an End of Project report which indicates that two out of three staff trained by donors in ART management during the pilot phase of ART implementation in for-profit health facilities no longer worked at the host organization [40].

Low sustainers and Non-Sustainers cited several ART program sustainability barriers related to workforce motivation constraints. Staff expressed dissatisfaction with the reward systems offered for coping with heavy workloads brought on by ART scale-up. Staff perceived their salaries to be low and the terms of service as a disincentive to long-term commitment and service on the ART program.

Although low salary levels were reported across all cases, HS-001 and HS-002 reported that they devised deliberate strategies to retain their ART-proficient staff. Attendance of training workshops by program staff was identified as an incentive that provided supplemental income in the form of per-diem allowances especially if these trainings were off-site. Participants reported adopting incentives to boost staff morale through payment of a lunch allowance or provision of meals on ART clinic days which were described as having high workloads.

What distinguished the cases, with regard to insufficient ART workforce remuneration, were the strategies devised by the ART program leadership to address this disincentive. High Sustainers reported a range of motivation strategies compared to Low and Non sustainers who didn’t report similar approaches.

## Broader environment factors

### Facilitators of ART program sustainability relating to the broader environment

#### External support environments and partnerships

HS-001 and HS-002 indicated that they derived program continuation support from other ART-providing organizations with whom they were allied. For instance, HS-002 sought support from other faith-based ART providers. The specific nature of support cited by interviewees includes; the borrowing of ARV drugs or specific drug combinations whenever they experienced stock outs and having patient referral in-lets and out-lets.

*“Some times when we experience drug stock outs, we borrow drugs from our sister providers in the faith-based fraternity and we replace their drugs when we replenish our stock”* [Interviewee 3, HS-002].

Both HS-001 and HS-002 reported long-standing external program champions who continued to mobilize resources to sustain their ART program over a period of more than 10 years. There was a concurrence between interviewee data and documentary review evidence in showing that the ART programs in both cases were founded by external champions who continued to mobilize resources to sustain HIV services even after returning to their home countries.

### Barriers to ART program sustainability relating to the broader environment

Our findings suggest that the local setting and area context or where a health facility is allocated influenced ART program sustainment outcomes.

#### Rural setting and associated local infrastructure barriers

Participants from both NS-001, NS-002 and LS-002 reported barriers to program sustainability related to their rural setting and its associated infrastructure constraints. They described their locations as too remote for the logistics chain to supply. NS-002 reported that whenever they experienced ARV drug stock outs, they were unable to borrow the drugs from an affiliated provider in the same district on account of the poor road network linking them. Participants from LS-001 perceived the geographic isolation of the health center and the long distance to the nearest referral and ARV re-supply lines as a barrier to ART program sustainment.

*“A sister health facility in our district was willing to share ARV drugs with us but the road to that health facility is very poor. In fact, in comparison, Kampala (the capital) is near… Kampala may be further but it is cheaper to get there”* [Interviewee 1, NS-002].

Participants from LS-001 indicated that electricity supply to their health center was unstable and impeded ART laboratory investigations and increased operational costs as it necessitated frequent use of a generator which was costly and affected patient satisfaction with the quality of ART services offered.

*“The electricity in this area is on and off. We always have to rely on a generator which uses fuel and as you can see this is not a rich health center. Sometimes, you are running laboratory tests and the electricity goes off and there is no fuel in the generator”* [Interviewee 3, NS-001].

## Discussion

We found several distinguishing features between health facilities which had the highest ART sustainability scores (High sustainers) and those with the lowest scores (low sustainers) and health facilities which didn’t sustain ART(Non sustainers). The most important distinguishing feature were factors in the internal organizational context of the cases. High Sustainers reported having an internal program champion, stable program leadership of at least 7 years, robust ART program reporting systems and long-standing external champions. The finding that the organizational culture and climate of the implementing provider differentiates between agencies which sustain interventions from those which don’t is widely supported in the literature [41–43].

There was concurrence in the barriers cited across the Low and Non-Sustainer categories which included attrition of ART-proficient staff, irregular and insufficient supply of ART commodities and absence of internal and external program champions. The degree of similarity in sustainability barriers and attributes cited across the cases that made up the Low Sustainer and Non-sustainer categories was high. This may suggest a convergence around the factors that detract from ART program sustainability that transcends case categorizations.

The case studies suggest that ART program sustainability objectives for for-profit providers were distinguished from other types of health facilities in Uganda as they were reported to be dependent on these programs being able to generate a profit in relation to provider investment. The findings demonstrate that the availability of donor support, in form of free supplies of ARV drugs, laboratory equipment and staff training, did not guarantee long-term program sustainability in for-profit providers. This finding agrees with previous studies which found that alignment of the intervention and organization’s mission influence sustainability outcomes [22, 44, 45]. In the six cases, we found that continued delivery of ART programs was donor-dependent and was influenced by factors external to the providers. Program continuation depended on meeting donor criteria and performance targets which were important drivers of sustained ART delivery. We observed a dependence by providers on time-limited funding to sustain their rapidly expanded ART programs. The study findings add to mounting calls for increasing local-ownership of HIV service delivery [4, 5, 7]. The finding that organizations are affected by external constraints and dynamics is well supported in the literature [46–48].

### The dynamic interactions driving ART program sustainability

A major finding of this study is that although barriers to ART program sustainment were cited independently by providers, our analysis revealed a dynamic interaction in these drivers. For instance, for three of the cases where profit-making was the overriding organizational goal, it’s plausible this orientation could have influenced their human resource management choices such as length of tenure and salary scales for the ART workforce which in turn affected retention and staffing strength. It emerged that ARV drug stock outs were partly a result of low staffing capacity which hampered the updating of ART registers which are used as the basis for ART commodities forecasts and requisitions to Uganda’s national medicines supplier.

ART workforce inputs such as staffing numbers and motivation influenced the ability of providers to deliver on ART program reporting mandates which in turn affected successor-grant prospects with funders. Low and Non sustainers were relatively constrained in meeting donor criteria of growing and retaining patient loads due to having lower internal capacity which resulted in less satisfactory client experiences characterized by drug stock outs, long waiting times and low patient follow-up capacities. With regard to the High Sustainers, we found that maintaining robust internal ART monitoring and evaluation systems attracted additional funding from the external environment and that this was in turn dependent on the organizational climate and culture of the host agency. The role of internal program champions and leaders was highlighted as influential in fostering ART program continuation and could have interacted with other sustainability drivers. An interaction in the factors affecting long-term implementation of interventions has been observed in previous studies [21, 55]. A dynamic interaction of ART program sustainability drivers emerged in the analyses involving diverse stakeholders namely; providers, patients, external funders and the local ARV drugs supply chain. A broader systems thinking has been called for in the literature on health service delivery in resource-constrained settings and our findings provide further empirical support for this approach [19, 46].

### Implications for Health Systems strengthening

The findings from the cases we examined suggest that ART program sustainability strategies and contexts in Uganda are distinguished by the size of the health facility. We found that over the last ten years, larger and established hospitals were able to attract multiple grants for ART delivery from funders compared to smaller and less-established health facilities. The prioritization of ART program evaluation by High sustainers, was a key distinguishing feature as it enabled them to demonstrate success to external funders. Smaller health facilities reported barriers in keeping up with their ART program evaluation mandates due to internal capacity constraints such as having inadequate staff and physical space. From a demand perspective, compared to large hospitals, patients were not attracted by a narrower ‘menu’ of HIV services, weaker patient follow-up capacity, longer waiting times due to fewer staff, more frequent drug stock-outs and unreliable electricity supply. This finding agrees with previous studies which have found that health facility characteristics affect patient retention rates on ART programs [52–54]. Previous studies have found that ‘mature’ organizations have lower HIV treatment unit costs and enjoyed economies of scale accruing from large patient volumes compared to less established health facilities [49].

Our paper illuminates barriers to realizing the ART scale-up goals in Uganda. This is especially with regard to for-profit providers who are an important part of the service delivery infrastructure and alleviate the over-burdened public sector. We note that private–for-profit health facilities constitute half of all health facilities in Uganda [50]. Part of the solution suggested by for-profit providers was that salary top-ups be provided to their ART workforce for the extra workload brought on by ART scale-up at their clinics - a proposal earlier suggested by Biesma et al. [51].

Our findings reveal capacity constraints in routine ART program reporting among for-profit providers which is suggestive of a need for interventions to strengthen capacity in program reporting, a critical aspect that impacts on the ARV supply chain, patient outcomes and successor grants from funders. A study by Kyayise et al. (2008) found similar constraints in for-profit HIV service providers in Uganda [50].

Our findings are suggestive of the kind of health facilities which are more likely to sustain ART programs and the organizational and environmental contexts that are conducive for the long-term sustainability of ART programs in Uganda. By describing the characteristics of health facilities which have been successful in sustaining ART, following a post-implementation phase in Uganda, our study contributes empirical evidence that is relevant to funders of ART programs in resource-limited settings.

### Implications for country ownership of ART service delivery in Uganda

The study findings suggest a dependence by health facilities in Uganda on external donor support for ART service delivery. An increased role of the Uganda government in ART service delivery and long-term sustainability is imperative. A gradual and phased increase in domestic budget support to ART service delivery is called for. Increasing government contribution to the cost of procurement of ART commodities through domestic resource mobilization could be a good step in promoting country ownership of HIV programs in Uganda which is a topical subject in the literature [4–7].

We found that for-profit providers were relatively constrained in sustaining ART programs. Policies and programs targeted at supporting PFPs are critical to continued ART scale-up in Uganda. Government service purchase agreements with for-profit providers or seconding government-salaried health workers to PFPs with high patient volumes, especially those located in rural settings, or during designated peak periods such as ART Clinic days, is worthy of consideration. In addition, training programs targeting ART Clinic mangers in the areas of human resources for health (HRH) and program reporting could enhance program sustainability outcomes. The Ministry of Health’s ART monitoring Unit needs to be strengthened to enable it deliver on its mandate of monitoring ART service delivery all over the country. Strengthening the program monitoring function of this unit through the development of early warning systems and research to identify health facilities that need interventions could go a long way in improving ART program sustainability outcomes.

Consideration of the program sustainability strategies elicited by the study and the barriers identified, by ART program managers and planners in Uganda and other resource-constrained settings could improve long-term sustainability outcomes of ART programs and contribute to efforts to realize the global health agenda of universal access to HIV treatment.

### Limitations

Some limitations are important to acknowledge. Given that a significant amount of time had elapsed since ART was piloted at participating health facilities, recall bias could have been a shortcoming even when we relied on multiple respondents per case and the triangulation of data sources. Because of a complex dynamic interaction between organizational and environmental facilitators and barriers to ART program sustainability, the direction of causality was at times difficult to distinguish. For instance, maintaining robust monitoring and evaluation systems for ART programs was associated with attracting additional donor funding. Conversely, concerted donor policies over the study period could have influenced the prioritization of ART program evaluation by health facilities in their bid to ensure sustained external grants. In this study, we interrogated the sustainability of ART programs in health facilities in Uganda from the perspective of the providing organizations. Additional interviews with policy makers and the community could have enabled more diverse perspectives on the study findings.

## Conclusion

We found that ART program sustainability was embedded in a complex system involving dynamic interactions between internal (program champion, staffing strength, program evaluation) and external (donors, ARVs supply chain, patient demand) drivers. In the cases we examined, ART program sustainability contexts and strategies were distinguished by the size of the health facility and having a private-for-profit orientation. Our study highlights the influence of the framework by Shediac-Rizkallah & Bone [20] in illuminating the complex and multi-faced nature of health program sustainability.

The study has implications for health system strengthening for ART scale-up in Uganda and other resource-limited settings.

## References

[1] UNAIDS. 90-90-90 An ambitious target to end the AIDS epidemic. 2015. from http://www.unaids.org/sites/default/files/media_asset/90-90-90_en_0.pdf. Accessed 18 Jan 2016.

[2] UN (2015). Transforming Our World: The 2030 Agenda for Global Action.

[3] WHO. Consolidated guidelines on the use of antiretroviral drugs for treating and preventing HIV infection: what’s new. Geneva; 2015. from http://www.who.int/hiv/topics/treatment/en/. Accessed 17 Jan 2016.

[4] Piot P, Abdool Karim SS, Hecht R, Legido-Quigley H, Buse K, Stover J, Resch S, Ryckman T, Møgedal S, Dybul M, Goosby E (2015). Defeating AIDS—advancing global health. Lancet.

[5] Vermund SH, Sidat M, Weil LF, Tique JA, Moon TD, Ciampa PJ (2012). Transitioning HIV care and treatment programs in southern Africa to full local management. AIDS (London, England).

[6] Merson MH, Curran JW, Griffith CH, Ragunanthan B (2012). The President’s Emergency Plan For AIDS Relief: from successes of the emergency response to challenges of sustainable action. Health Aff.

[7] Palen J, El-Sadr W, Phoya A, Imtiaz R, Einterz R, Quain E, Blandford J, Bouey P, Lion A (2012). PEPFAR, health system strengthening, and promoting sustainability and country ownership. J Acquir Immune Defic Syndr.

[8] El-Sadr WM, Holmes CB, Mugyenyi P, Thirumurthy H, Ellerbrock T, Ferris R, Sanne I, Asiimwe A, Hirnschall G, Nkambule RN, Stabinski L. Scale-up of HIV treatment through PEPFAR: a historic public health achievement. J Acquir Immune Defic Syndr (1999). 2012;15:60(Suppl 3):S96. 10.1097/QAI.0b013e31825eb27b PMC3445041 22797746

[9] Windisch R, Waiswa P, Neuhann F, Scheibe F, de Savigny D (2011). Scaling up antiretroviral therapy in Uganda: using supply chain management to appraise health systems strengthening. Global Health.

[10] PEPFAR. Uganda Country Operational Plan 2014. Retrieved 14 Mar 2016 from http://www.pepfar.gov/countries/cop/240159.htm. Accessed 11 Oct 2016.

[11] Oomman N, Bernstein M, Rosenzweig S (2007). Following the funding for HIV/AIDS: a comparative analysis of the funding practices of PEPFAR the Global Fund and World Bank MAP in Mozambique Uganda and Zambia.

[12] Barker PM, McCannon CJ, Mehta N, Green C, Youngleson MS, Yarrow J, Bennett B, Berwick DM (2007). Strategies for the scale-up of antiretroviral therapy in South Africa through health system optimization. J Infect Dis.

[13] Harries AD, Makombe SD, Libamba E, Schouten EJ (2011). Why did the scale-up of HIV treatment work?: a case example from Malawi. J Acquir Immune Defic Syndr.

[14] Assefa Y, Jerene D, Lulseged S, Ooms G, Van Damme W (2009). Rapid scale-up of antiretroviral treatment in Ethiopia: successes and system-wide effects. PLoS Med.

[15] Van Oosterhout JJ, Kumwenda JK, Hartung T, Mhango B, Zijlstra EE (2007). Can the initial success of the Malawi ART scale-up programme be sustained? The example of Queen Elizabeth Central Hospital, Blantyre. AIDS Care.

[16] Stringer JS, Zulu I, Levy J, Stringer EM, Mwango A, Chi BH, Mtonga V, Reid S, Cantrell RA, Bulterys M, Saag MS (2006). Rapid scale-up of antiretroviral therapy at primary care sites in Zambia: feasibility and early outcomes. JAMA.

[17] Mutevedzi PC, Lessells RJ, Heller T, Bärnighausen T, Cooke GS, Newell ML (2010). Scale-up of a decentralized HIV treatment programme in rural KwaZulu-Natal, South Africa: does rapid expansion affect patient outcomes?. Bull World Health Organ.

[18] Keiser O, Chi BH, Gsponer T, Boulle A, Orrell C, Phiri S, Maxwell N, Maskew M, Prozesky H, Fox MP, Westfall A (2011). Outcomes of antiretroviral treatment in programmes with and without routine viral load monitoring in Southern Africa. AIDS (London, England).

[19] Gilson L (2012). Introduction to health policy and systems research. Health policy and systems research: a methodology reader.

[20] Shediac-Rizkallah MC, Bone LR (1998). Planning for the sustainability of community-based health programs: conceptual frameworks and future directions for research, practice and policy. Health Educ Res.

[21] Stirman SW, Kimberly J, Cook N, Calloway A, Castro F, Charns M (2012). The sustainability of new programs and innovations: a review of the empirical literature and recommendations for future research. Implement Sci.

[22] Scheirer MA, Dearing JW (2011). An agenda for research on the sustainability of public health programs. Am J Public Health.

[23] Goodman RM, McLeroy KR, Steckler AB, Hoyle RH (1993). Development of level of institutionalization scales for health promotion programs. Health Educ Behav.

[24] Johnson K, Hays C, Center H, Daley C (2004). Building capacity and sustainable prevention innovations: A sustainability planning model. Eval Program Plann.

[25] Proctor E, Silmere H, Raghavan R, Hovmand P, Aarons G, Bunger A, Griffey R, Hensley M (2011). Outcomes for implementation research: conceptual distinctions, measurement challenges, and research agenda. Adm Policy Ment Health Ment Health Serv Res.

[26] Savaya R, Spiro S, Elran-Barak R (2008). Sustainability of social programs: a comparative case study analysis. Am J Eval..

[27] Wright DB (2009). Care in the country: a historical case study of long-term sustainability in 4 rural health centers. Am J Public Health.

[28] LaPelle NR, Zapka J, Ockene JK (2006). Sustainability of public health programs: the example of tobacco treatment services in Massachusetts. Am J Public Health.

[29] Stolldorf DP. The sustainability of innovations in hospitals: A look at rapid response teams (Doctoral dissertation, THE UNIVERSITY OF NORTH CAROLINA AT CHAPEL HILL). 2013. from http://dc.lib.unc.edu/cdm/ref/collection/etd/id/5626. Accessed 27 Mar 2015.

[30] Zakumumpa H, Galarraga O, Bennett S, Ssengooba F. A Comparison of the Institutionalization of ART programs in four categories of health facilities in Uganda, 8^TH^ International AIDS Society Conference on HIV Pathogenesis, Treatment and Prevention. 2015. Abstract No. WEPED885. Abstract Book, Page 374. Retrieved 27^th^ October 2015 from http://www.ias2015.org/WebContent/File/IAS_2015__MED2.pdf. Accessed 11 Oct 2016.

[31] Zakumumpa H, Bennett S, Ssengooba F. Human resources for health strategies for the long-term sustainment of ART interventions: Findings from a national survey of health facilities in Uganda. Population Association of America (PAA 2016) Annual Meeting 2016. 2016. Abstract No.P1-98. from https://paa.confex.com/paa/2016/meetingapp.cgi/Paper/5997. Accessed 5 Aug 2016.

[32] Brinkerhoff R. The success case method: Find out quickly what’s working and what’s not. Berrett-Koehler Publishers; 2003. https://www.bkconnection.com/books/title/the-success-case-method.

[33] Coryn CL, Schröter DC, Hanssen CE (2009). Adding a time-series design element to the success case method to improve methodological rigor an application for nonprofit program evaluation. Am J Eval.

[34] Chan AK, Ford D, Namata H, Muzambi M, Nkhata MJ, Abongomera G, Mambule I, South A, Revill P, Grundy C, Mabugu T (2014). The Lablite project: A cross-sectional mapping survey of decentralized HIV service provision in Malawi, Uganda and Zimbabwe. BMC Health Serv Res.

[35] Luboga SA, Stover B, Lim TW, Makumbi F, Kiwanuka N, Lubega F, Mukooyo E, Hurley EK, Borse N, Wood A, Bernhardt J. Did PEPFAR investments result in health system strengthening? A retrospective longitudinal study measuring non-HIV health service utilization at the district level. Health policy and planning. 2016;czw009. 10.1093/heapol/czw009 PMC4977428 27017824

[36] Ssengooba FP. Performance-based contracting: case-study for non-profit hospitals in Uganda. Doctoral thesis, London School of Hygiene Tropical Medicine. 2010. from https://researchonline.lshtm.ac.uk/682436/1/550380.pdf. Accessed 25 July 2015.

[37] Yin RK. Case study research: Design and methods. Sage publications; 2013. https://uk.sagepub.com/en-gb/asi/case-study-research/book237921.

[38] Ivankova NV, Creswell JW, Stick SL (2006). Using mixed-methods sequential explanatory design: From theory to practice. Field Methods.

[39] Miles MB, Huberman AM. Qualitative data analysis: An expanded sourcebook. Thousands Oaks. https://uk.sagepub.com/en-gb/asi/qualitative-data-analysis/book239534.

[40] USAID. The Health Initiatives for the Private Sector (HIPS) Project. Final Evaluation Report, January 2013. http://pdf.usaid.gov/pdf_docs/Pdacu928.pdf.

[41] Schell SF, Luke DA, Schooley MW, Elliott MB, Herbers SH, Mueller NB, Bunger AC (2013). Public health program capacity for sustainability: a new framework. Implement Sci.

[42] Glisson C, Schoenwald SK, Kelleher K, Landsverk J, Hoagwood KE, Mayberg S, Green P (2008). Research Network on Youth Mental Health. Therapist turnover and new program sustainability in mental health clinics as a function of organizational culture, climate, and service structure. Adm Policy Ment Health Ment Health Serv Res.

[43] Goodson P, Smith MM, Evans A, Meyer B, Gottlieb NH (2001). Maintaining prevention in practice: survival of PPIP in primary care settings. Am J Prev Med.

[44] Bowman CC, Sobo EJ, Asch SM, Gifford AL, HIV/Hepatitis Quality Enhancement Research Initiative (2008). Measuring persistence of implementation: QUERI Series. Implement Sci.

[45] Harvey G (2006). Exploring program sustainability: identifying factors in two educational initiatives in Victoria. Eval J Australasia.

[46] Gruen RL, Elliott JH, Nolan ML, Lawton PD, Parkhill A, McLaren CJ, Lavis JN (2008). Sustainability science: an integrated approach for health-programme planning. Lancet.

[47] Bossert TJ (1990). Can they get along without us? Sustainability of donor-supported health projects in Central America and Africa. Soc Sci Med.

[48] Pfeffer J, Salancik GR. The external control of organizations: A resource dependence perspective. Stanford University Press; 2003. http://www.sup.org/books/title/?id=5889.

[49] Menzies NA, Berruti AA, Berzon R, Filler S, Ferris R, Ellerbrock TV, Blandford JM (2011). The cost of providing comprehensive HIV treatment in PEPFAR-supported programs. AIDS (London, England).

[50] Kyayise A, Kyeyagalire R, Livesley N, Kirunda I, Tumwesigye B. Private-for-profit HIV/AIDS care in Uganda: an assessment. 2008. from http://www.urc-chs.com/sites/default/files/UgandaPFPassessmentfulltechnicalreport_USltr.pdf. Accessed 31 Jan 2016.

[51] Biesma RG, Brugha R, Harmer A, Walsh A, Spicer N, Walt G (2009). The effects of global health initiatives on country health systems: a review of the evidence from HIV/AIDS control. Health Policy Plan.

[52] Miller CM, Ketlhapile M, Rybasack‐Smith H, Rosen S (2010). Why are antiretroviral treatment patients lost to follow‐up? A qualitative study from South Africa. Tropical Med Int Health.

[53] Hardon AP, Akurut D, Comoro C, Ekezie C, Irunde HF, Gerrits T, Kglatwane J, Kinsman J, Kwasa R, Maridadi J, Moroka TM (2007). Hunger, waiting time and transport costs: time to confront challenges to ART adherence in Africa. AIDS Care.

[54] Assefa Y, Lynen L, Wouters E, Rasschaert F, Peeters K, Van Damme W (2014). How to improve patient retention in an antiretroviral treatment program in Ethiopia: a mixed-methods study. BMC Health Serv Res.

[55] Durlak JA, DuPre EP (2008). Implementation matters: A review of research on the influence of implementation on program outcomes and the factors affecting implementation. Am J Community Psychol.

